# Comparative genomic and functional analyses: unearthing the diversity and specificity of nematicidal factors in *Pseudomonas putida* strain 1A00316

**DOI:** 10.1038/srep29211

**Published:** 2016-07-07

**Authors:** Jing Guo, Xueping Jing, Wen-Lei Peng, Qiyu Nie, Yile Zhai, Zongze Shao, Longyu Zheng, Minmin Cai, Guangyu Li, Huaiyu Zuo, Zhitao Zhang, Rui-Ru Wang, Dian Huang, Wanli Cheng, Ziniu Yu, Ling-Ling Chen, Jibin Zhang

**Affiliations:** 1 State Key Laboratory of Agricultural Microbiology and National Engineering Research Center of Microbe Pesticides, College of Life Science and Technology, Huazhong Agricultural University, Wuhan 430070, Hubei, China; 2 Agricultural Bioinformatics Key Laboratory of Hubei Province, College of Informatics, Huazhong Agricultural University, Wuhan 430070, Hubei, China; 3 Key Laboratory of Marine Biogenetic Resources, Third Institute of Oceanography, State Oceanic Administration, Xiamen 361005, Fujian, China

## Abstract

We isolated *Pseudomonas putida* (*P. putida*) strain 1A00316 from Antarctica. This bacterium has a high efficiency against *Meloidogyne incognita* (*M. incognita*) *in vitro* and under greenhouse conditions. The complete genome of *P. putida* 1A00316 was sequenced using PacBio single molecule real-time (SMRT) technology. A comparative genomic analysis of 16 *Pseudomonas* strains revealed that although *P. putida* 1A00316 belonged to *P. putida*, it was phenotypically more similar to nematicidal *Pseudomonas fluorescens* (*P. fluorescens*) strains. We characterized the diversity and specificity of nematicidal factors in *P. putida* 1A00316 with comparative genomics and functional analysis, and found that *P. putida* 1A00316 has diverse nematicidal factors including protein alkaline metalloproteinase AprA and two secondary metabolites, hydrogen cyanide and cyclo-(l-isoleucyl-l-proline). We show for the first time that cyclo-(l-isoleucyl-l-proline) exhibit nematicidal activity in *P. putida*. Interestingly, our study had not detected common nematicidal factors such as 2,4-diacetylphloroglucinol (2,4-DAPG) and pyrrolnitrin in *P. putida* 1A00316. The results of the present study reveal the diversity and specificity of nematicidal factors in *P. putida* strain 1A00316.

Members of the genus *Pseudomonas* display remarkable physiological and metabolic versatility, which enables them to colonize diverse terrestrial and aquatic habitats such as soil^1^, plants^2^, polluted creeks^3^, and fresh water^4^. Among species of the genus, *P. putida* has been isolated from many different niches and has the ability to survive in soils containing high concentrations of organic contaminants and heavy metals^5^. They can degrade a wide variety of chemicals, including many natural and synthetic compounds^6^. Some *P. putida* strains are plant growth-promoting rhizospheric and endophytic bacteria, which make them ideal for biocontrol^7^. However, the effects of *P. putida* strains on *Meloidogyne incognita* (*M. incognita*) have seldom been reported and the mechanisms by which they control *M. incognita* are unclear^8^.

*M. incognita* infection can cause serious plant parasitic diseases, which greatly limits agricultural productivity and quality^9^. *M. incognita* on cucumbers and tomatoes was mainly controlled by fumigants such as methyl bromide^10^, metal sodium, and 1,3-dichloropropene^11^. Although these chemical nematicides are effective, they have been withdrawn from use or restricted owing to serious environmental safety and public health concerns^12^. Accordingly, it has become necessary to identify novel and environmental friendly alternatives to control plant-parasitic nematode populations.

*Pseudomonas* species are ubiquitous in the natural world and produce a remarkable array of secondary metabolites active against agriculturally important plant diseases^13^. For example, some *P. fluorescens* strains can produce proteins and secondary metabolites that function as biocontrol factors to kill nematodes, such as 2,4-diacetylphloroglucinol (2,4-DAPG)^14^,^15^, alkaline metalloproteinase AprA^16^, pyrrolnitrin (Prn)^17^, and hydrogen cyanide (HCN)^17^. Loper *et al.* characterized the diversity and phylogeny of plant-associated *Pseudomonas* spp. involved in multitrophic interactions based on the comparative genomic study^18^. They found that ten *P. fluorescens* strains exhibited a diverse spectrum of traits involved in biological control and other multitrophic interactions with plants, microbes, and insects. However, that study of secondary metabolite biosynthesis did not include functional analyses of biocontrol activity. In addition, although some other comparative genome analysis of *Pseudomonas* strains have also been reported, they mainly focused on the *Pseudomonas* strains which degraded organic pollutant or some plant growth-promoting rhizobacteria (PGPR) in *Pseudomonas*^19^,^20^,^21^,^22^.

In this study, we report the complete genome sequence of *P. putida* 1A00316 isolated from Antarctica soil. The assembled genome sequence has been deposited in NCBI Refseq database under accession number CP014343. The bacterium inhibits *M. incognita in vitro* and under greenhouse conditions, where it reduces symptoms up to 71.67% on tomato caused by *M. incognita*^23^. We performed comparative genomic and phenotypic analyses of 16 strains within the *Pseudomonas* group (five *P. fluorescens*, two *P. protegens*, and nine *P. putida* strains), including the newly sequenced *P. putida* 1A00316. We observed a diverse spectrum of genomic features, evolutionary relationships, and virulence factors among these strains. We further identified and characterized the nematicidal factors in *P. putida* 1A00316, and explored their distribution within other *Pseudomonas* strains.

## Results and Discussion

### Genomic features of *Pseudomonas* strains

**Figure 1:**
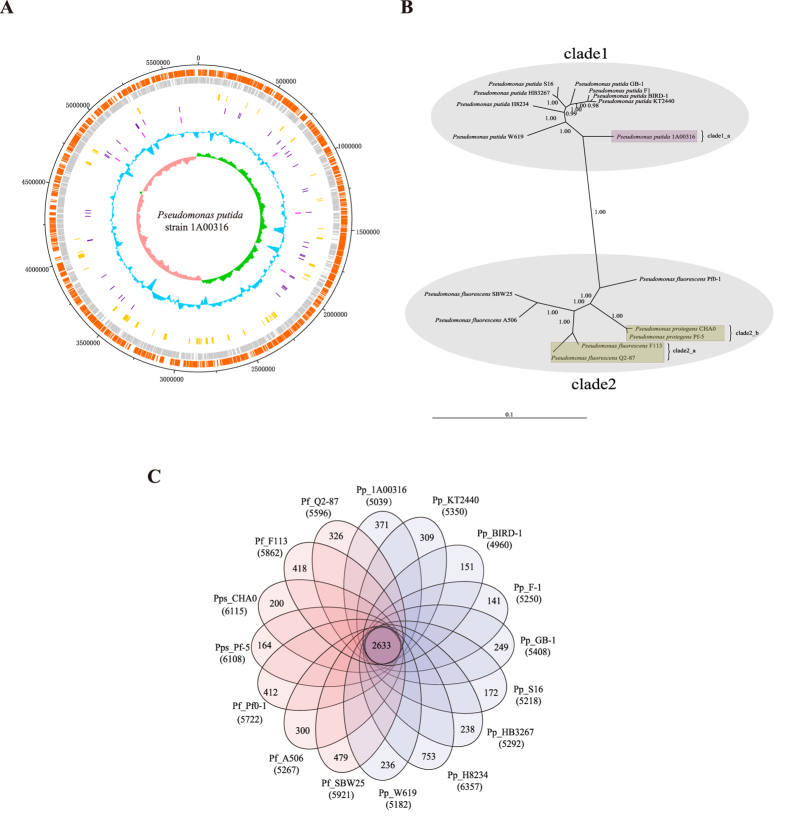
Genomic features and a comparative genomic analysis of *Pseudomonas* strains. (**A**) Circular plot of the *P. putida* 1A00316 chromosome. Circles are numbered from 1 (outermost) to 8 (innermost). Circle 1 represents the whole chromosome; Circles 2 and 3 show the locations of predicted CDSs on the positive and negative strands, respectively; Circle 4, genomic islands; Circle 5, tRNA genes; Circle 6, rRNA genes; Circle 7, %G + C; Circle 8, GC skew ((G−C)/(G + C)). (**B**) Phylogenetic tree depicting the relationships among 16 *Pseudomonas* strains. The values shown at interior nodes represent clade credibility, which is the likelihood of the clade based on the posterior probability values generated using MrBayes. (**C**) Genomic diversity of 16 *Pseudomonas* strains. Each strain is represented by an oval. The number of orthologous coding sequences (CDSs) shared by all strains (i.e., the core genome) is shown in the center. Overlapping regions show the number of CDSs conserved only within the specified genomes. Numbers in non-overlapping portions show the number of CDSs unique to each strain. The total number of protein-coding genes within each genome is listed below the strain name.

The genomic features of *P. putida* 1A00316 and other 15 reference *Pseudomonas* strains are summarized in Table 1. The newly sequenced complete genome sequence of *P. putida* 1A00316 comprised a circular chromosome of 5,715,815 bp containing 5,039 protein-coding genes, 22 rRNA and 76 tRNA genes with an average G + C content of 64.4% (Table 1; Fig. 1A). Interestingly, *P. putida* 1A00316 had the highest GC content among the selected *Pseudomonas* strains. The genome size and number of protein-coding genes for these strains ranged from 5.72 ~ 7.07 Mb and 4,960 ~ 6,357 genes, respectively, demonstrating substantial strain-to-strain variation.

### Phylogenetic tree

We constructed a phylogenetic tree of the 16 *Pseudomonas* strains based on 16 S rRNA and five conserved genes (*dnaE*, *guaA*, *gyrB*, *recA*, and *rpoB*)^24^ (Fig. 1B). The 16 *Pseudomonas* strains clustered into two major clades. The *P. fluorescens* group clearly formed a single, large clade composed of four distinct sub-clades. *P. fluorescens* Q2–87 (producing 2,4-DAPG, which may have nematicidal activity)^25^ and nematicidal strain *P. fluorescens* F_113^26^ were in clade2_a, and clade2_b was composed of two other closely related nematicidal strains, *P. protegens* CHA0^14^,^15^,^16^ and Pf-5^27^. The other large clade was composed of nine *P. putida* strains and was clearly divided into two sub-clades. Clade1_a contained only *P. putida* 1A00316, indicating that it was distinct from other *P. putida* strains. We also observed similar patterns between the above-described phylogenetic tree and a maximum likelihood phylogeny inferred from 2,408 single-copy protein-coding genes conserved in these *Pseudomonas* strains (see Supplementary Figure S1).

### Analysis and comparison of virulence factors

To further explore the relationships among the *Pseudomonas* strains, we predicted virulence factors in each strain using the virulence factor database (VFDB)^28^ and performed a comprehensive comparative analysis of these factors. As summarized in Table 1, the number of virulence factors in strains of the *P. fluorescens* group (77–110) was generally higher than that in *P. putida* group (62–72), except for *P. putida* 1A00316 (92). We speculated that *P. putida* 1A00316 differs from other *P. putida* strains with respect to virulence metabolism and is more similar to *P. fluorescens* group. To evaluate this conjecture, we compared the types of virulence factors between *P. putida* 1A00316 and other *P. fluorescens* and *P. putida* strains. *P. putida* 1A00316 had 30 unique virulence factors with respect to *P. putida* strains, but only eleven unique virulence factors with respect to the nematicidal *P. fluorescens* strains. These results support our observation that at least with respect to virulence genes, *P. putida* 1A00316 is more similar to *P. fluorescens* than *P. putida* group.

### Identification and comparison of protein families

Using orthoMCL^29^, we identified a core genome containing 2,633 protein-coding genes shared among the 16 *Pseudomonas* strains (Fig. 1C). The core genome accounted for only 41–53% of the proteome of each strain, indicating a high degree of genomic diversity in the *Pseudomonas* group. To examine the genomic diversity among the nematicidal strains, we determined the size of the core genome and the pan-genome of four nematicidal strains (*P. putida* 1A00316, and *P. fluorescens* F_113 and two *P. protegens* strains), which contained 3,261 and 8,666 protein-coding genes, respectively. We also characterized the core genome and pan-genome of four additional strains, including the three nematicidal *P. fluorescens* strains and one randomly selected *P. putida* strain that lacks nematicidal ability. Their core genomes and pan-genomes contained 3,136 ~ 3,217 and 9,108 ~ 10,003 genes, respectively. These results demonstrated that the genomic complexity among the four nematicidal strains is less than that of other taxon combinations (not all strains in this group can kill *M. incognita*).

### *P. putida* 1A00316 contained a nematicidal factor encoding alkaline metalloproteinase AprA

*P. putida* 1A00316 exhibits strong inhibition against *M. incognita in vitro* and under greenhouse conditions^23^, while all of the other selected *P. putida* strains lack the ability to kill *M. incognita*. Thus, additional studies of this important bacterium, especially its nematicidal factors, are worthwhile. Several factors active against *M. incognita* have been detected in other nematicidal bacteria^26^,^27^,^30^,^31^,^32^, including Prn, 2,4-DAPG, HCN, and alkaline metalloproteinase AprA. To detect such factors and identify their functions in *P. putida* 1A00316, we performed a series of biological experiments and bioinformatics analyses.

**Figure 2:**
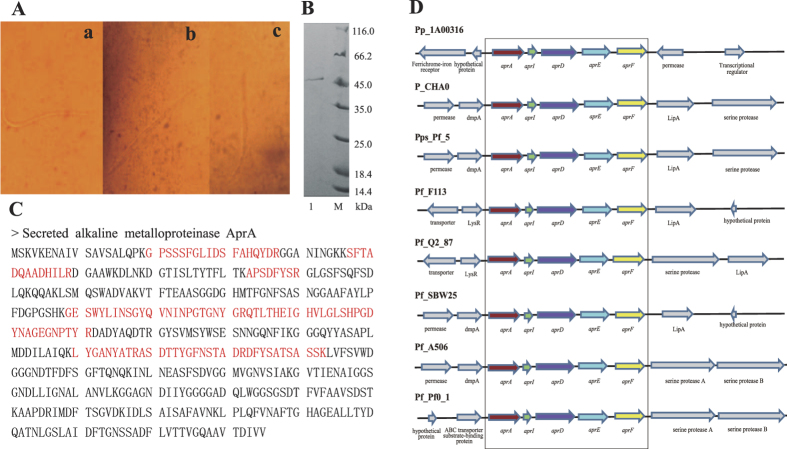
Characterization of the alkaline metalloproteinase AprA in *P. putida* 1A00316. (**A**) The light microscope results for *M. incognita* processed with the *P. putida* 1A00316 protein. ‘a’, ‘b’ and ‘c’ processed with the nematicidal protein for 0 h, 12 h and 24 h respectively. (**B**) The purified fraction exhibiting nematicidal activity after Sephadex G75 chromatography was assayed by SDS-PAGE. M represents the protein marker, line 1 represents the *P. putida* 1A00316 purified fraction exhibiting nematicidal activity after Sephadex G75 chromatography. (**C**) Protein bands detected by MALDI-TOF MS. The six red amino acid fragments were peptides, and can all be matched to a protein annotated alkaline metalloproteinase AprA. (**D**) *aprA* gene cluster in different *Pseudomonas* strains.

We examined the toxicity of the crude protein extract from *P. putida* 1A00316 against *M. incognita* under an inverted microscope. Initially, the cuticles and intestinal tissues of *M. incognita* were smooth and intact. A vacuole began to form and the intestinal tissue became ambiguous after 12 h, but the body wall was still integrated. After 24 h, the intestinal tissue disappeared completely (Fig. 2A). High temperature and proteinase K inactivated the preparations, confirming that the active fraction was a protein. The crude protein was then purified by ammonium sulfate precipitation, Sephadex G75 chromatography, cation-exchange CM52 chromatography and assayed for nematicidal activity and protein content. After the cation-exchange CM52 column step, we observed a mortality rate of the active fraction of 41.6%. SDS-PAGE showed a prominent band of approximately 48.0 kDa (Fig. 2B), in reasonable agreement with the previously determined molecular mass of 49.9 kDa for alkaline metalloprotease AprA from a different *P. fluorescens* strain^16^.

The 48-kDa band identified by SDS-PAGE was excised and analyzed by MALDI-TOF MS. Six peptides were detected for the protein, all of which matched to the gene encoding alkaline metalloproteinase AprA in *P. putida* 1A00316 genome (Fig. 2C). We detected the *aprA* gene cluster in all of the selected *P. fluorescens* strains, but only in *P. putida* 1A00316 within the *P. putida* group (Fig. 2D), suggesting that AprA protein may have an important function in this peculiar bacterium. The upstream and downstream genes of *aprA* gene cluster were conserved in the *P. fluorescens* strains, but were quite different in *P. putida* 1A00316. We detected genes encoding a ferrochrome-iron receptor and a hypothetical protein upstream of the *aprA* gene cluster in *P. putida* 1A00316, and genes encoding a permease and a transcriptional regulator downstream of the cluster. In addition to the comparative analysis of the genetic organization of the region surrounding *aprA* gene cluster, we compared AprA sequences between *P. putida* 1A00316 and the *P. fluorescens* group strains. Based on the sequence alignment, the identity of AprA protein between *P. putida* 1A00316 and four nematicidal strains was 70%, and the ZnMc_serralysin-like structural domain and active sites were highly conserved (Supplementary Figure S2). Mutations in AprA and GacS/GacA signal transduction pathway lead to a sharp decline in nematicidal activity in *P. fluorescens* CHA0^16^, indicating that both are involved in the process of killing *M. incognita*. Based on the above results, we proved that AprA protein in *P. putida* 1A00316 contributes to the biocontrol of *M. incognita*.

### Comparative analysis of small-molecule nematicidal factors in *Pseudomonas* strains

**Figure 3:**
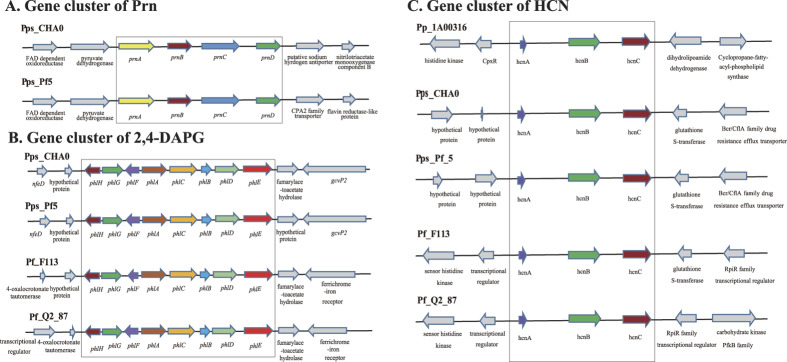
Genetic organization of the region containing small-molecular-weight nematicidal factors. (**A**) *Prn* gene cluster. (**B**) Cluster of genes encoding 2,4-DAPG. (**C**) HCN gene cluster. The genes included in the box are the synthetic gene cluster related to each nematicidal factor, while genes outside the box are located upstream/downstream of each gene cluster.

**Figure 4:**
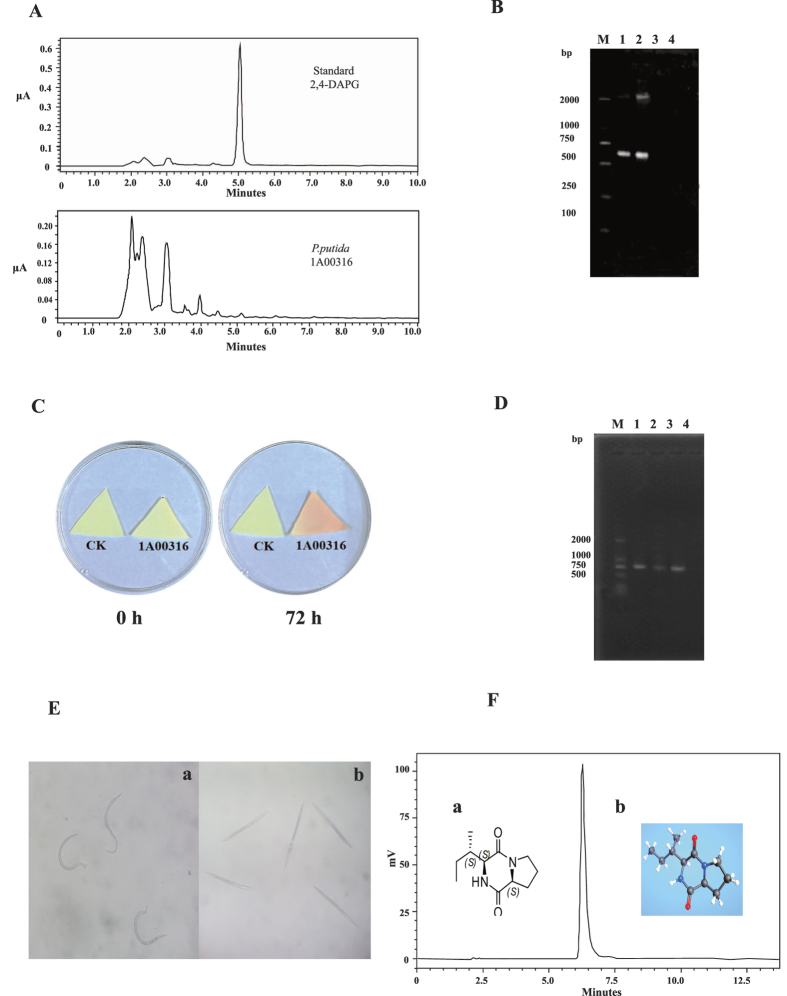
Experimental analysis of small molecule of nematicidal factors in *P. putida* 1A00316. (**A**) HPLC chromatogram of standard 2,4-DAPG and crude extracts of *P. putida* 1A00316 fermentation broth. (**B**) PCR amplification of the *phlD* gene fragment. M represents DL 2000, lines 1–3 represent the DNA of *P. fluorescens* CHA0, *P. fluorescens* Pf-5, and *P. putida* 1A00316, respectively, and line 4 is the water control. (**C**) HCN detection with biochemical methods. HCN produced by *P. putida* 1A00316 in microtiter plates with indicator paper at 0 and 72 h. Yellow paper indicated that *P. putida* 1A00316 could not produce HCN, while orange indicated that it could produce HCN. (**D**) PCR amplification of the *hcnB* gene fragment. M represents the marker of DL 2000, lines 1–3 represent the DNA of *P. fluorescens* CHA0, *P. fluorescens* Pf-5, and *P. putida* 1A00316, respectively, and line 4 is the water control. (**E**) The light microscopy results for *M. incognita* treated with the small metabolite cyclo-(l-Ile-l-Pro) produced by *P. putida* 1A00316. ‘a’ and ‘b’ treated by cyclo-(l-Ile-l-Pro) for 0 h and 24 h respectively. (**F**) Structures of cyclo-(l-Ile-l-Pro) and HPLC results for purified cyclo-(l-Ile-l-Pro) from *P. putida* 1A00316. ‘a’ and ‘b’ represent the two-dimensional and three-dimensional structures of cyclo-(l-Ile-l-Pro), respectively.

Nematicidal strains typically contain several biocontrol factors. The death of *M. incognita* is usually not caused by a single factor, but by the synergistic effects of several factors^15^,^33^. AprA was not the only nematicidal factor in *P. putida* 1A00316, and we believe that small molecular metabolites also act as biocontrol factors, such as in other nematicidal bacteria^17^. Figure 3 summarizes the diversity of small molecular nematicidal factors in the selected bacterial strains examined in this study. We only observed the biosynthetic *Prn* gene cluster in two nematicidal strains, *P. protegens* CHA0 and Pf-5 (Fig. 3A), and detected 2,4-DAPG biosynthesis genes in three nematicidal *P. fluorescens* strains and *P. fluorescens* Q2–87, but not in *P. putida* 1A00316 (Fig. 3B). We further compared the retention times of standard 2,4-DAPG and crude extract from the culture supernatant of *P. putida* 1A00316 by high-performance liquid chromatography (HPLC) (Fig. 4A), and confirmed the absence of 2,4-DAPG. We further PCR-amplified the locus of *phlD* gene in *P. putida* 1A00316, *P. fluorescens* CHA0, and *P. protegens* Pf-5^34^ (Fig. 4B). As shown in Fig. 4A,B, we were unable to amplify *phlD* in *P. putida* 1A00316 and had not detected 2,4-DAPG in the crude extract of this bacterium. Based on these analyses, we determined that *P. putida* 1A00316 could not produce 2,4-DAPG. Accordingly, 2,4-DAPG was not associated with nematicidal activity in this strain.

However, we did detect *hcn* biosynthetic genes in the *P. putida* 1A00316 genome, and these loci were virtually identical in sequence and organization to those of four other nematicidal strains (Fig. 3C). The sequences of genes located upstream and downstream of the *hcn* gene cluster in four *P. fluorescens* strains were highly similar or identical but differed from those present in *P. putida* 1A00316, in which the two-component system response regulator CpxR and a signal transduction histidine kinase were located upstream and dihydrolipoamide hydrogenase and cyclopropane-fatty-acyl-phospholipid synthase were downstream of the *hcn* gene cluster. We also confirmed the presence of *hcn* gene cluster in *P. putida* 1A00316 genome using a biochemical experiment. As shown in Fig. 4C,D, we detected a color change from yellow to orange on the picric acid indicator paper after 72 h, indicating the production of HCN by 1A00316, and we successfully PCR-amplified the gene from the strain. According to previous studies, HCN is toxic to *Caenorhabditis elegans*^35^ and *M. incognita*^36^, suggesting that HCN contributes to the biocontrol of *M. incognita* in *P. putida* 1A00316 as well as other nematicidal *Pseudomonas* strains.

### A nematicidal factor in *P. putida* 1A00316 is a small molecular metabolite, cyclo-(l-Ile-l-Pro)

We observed substantial nematicidal activity in *n*-butyl alcohol extracts from the culture filtrate of *P. putida* 1A00316 (Fig. 4E), and the samples were loaded for HPLC. Finally, the chromatogram of P24-P36 was eluted and we detected a single peak at 210 nm with a retention time of 6.0 min (Fig. 4F).

Based on an electron ionization mass spectrometry (EI/MS) analysis, we confirmed that the compound had a molecular ion at m/z 211.1449 with a formula of C_11_H_18_N_2_O_2_. We also determined its two-dimensional and three-dimensional structures (Fig. 4F). Based on the characteristics of ^1^H nuclear magnetic resonance (NMR) spectrum and other two-dimensional NMR spectra (Table 2), the compound was cyclic dipeptide cyclo-(l-Ile-l-Pro) (Fig. 4F). Cyclic dipeptides (also known as 2,5-dioxopiperazines) are formed by two amino acids with peptide bond cyclization, which are the smallest cyclo(peptide) in nature and can be formed by alpha amino acids (often l-amino acids) to generate a relatively stable six-member ring, and acts as an important pharmacophore in medicinal chemistry. A variety of cyclic dipeptides with different compositions and structures have been discovered. Generally, cyclo(dipeptide)s and their corresponding synthases differ among species. For example, *Streptomyces noursei*^37^, *Mycobacterium tuberculosis* H37Rv^38^, and *Staphylococcus haemolyticus* JCSC1435^39^ have cyclo-(l-Phe-l-Leu) (synthase, gi: 323463044), cyclo-(l-Tyr-l-Tyr) (synthase, gi: 614130859), and cyclo-(l-Leu-l-Leu) (synthase, gi: 123658993), respectively. Interestingly, though some species share the same cyclo(dipeptide)s, their biosynthetic pathways are highly different^39^,^40^,^41^. We compared the synthases responsible for cyclo(dipeptide)s in other bacteria to all the encoding genes of *P. putida* 1A00316, and observed a very low amino acid sequence identity, suggesting that the synthases in *P. putida* 1A00316 are unique. Although the compound cyclo-(l-Ile-l-Pro) has been reported previously and shows antimicrobial activity against *Vibrio anguillarum* (minimal inhibitory concentration: 0.03 to 0.03 μg/mL)^42^, its nematicidal activity had not reported previously.

To comprehensively analyze nematicidal activity, we treated *M. incognita* with various concentrations of cyclo-(l-Ile-l-Pro) (0.13, 0.33, and 0.50 mg/mL). As summarized in Table 3, as the cyclo-(l-Ile-l-Pro) concentration and incubation time increased, the mortality of stage 2 juveniles (J2) increased.

## Conclusions

*P. putida* strain 1A00316 was isolated from Antarctica and displayed distinct traits compared with other *P. putida* strains. In a comparative genomic analysis of 16 *Pseudomonas* genomes, we observed high diversity of nematicidal factors. *P. putida* 1A00316 and *P. fluorescens* F_113 had three nematicidal factors, *P. protegens* strains CHA0 and Pf-5 had five nematicidal factors, and the other strains did not have nematicidal factors. We have not detected common nematicidal factors such as 2,4-DAPG and Prn in *P. putida* 1A00316, but we detected the synthetic gene clusters encoding for HCN and AprA, which are related to nematicidal activity. The expression of these two factors was detected using biochemical methods, and nematicidal activity of AprA protein was confirmed. In addition, we isolated and characterized a new nematicidal factor cyclo-(l-Ile-l-Pro) in *P. putida* 1A00316. Our study combines a comparative genomic analysis with functional identification and active substance analyses to explore the diversity and specificity of nematicidal factors in *P. putida* strain 1A00316, and also provides a new nematicidal factor for the control of *M. incognita* population.

## Material and Methods

### *P. putida* 1A00316 genome sequence and CDS annotation

The *P. putida* 1A00316 genome was sequenced using the PacBio single molecule real-time (SMRT) technology (Wuhan Institute of Biotechnology, Wuhan, China)^43^. A 3 ~ 20 kb genomic DNA library was prepared suitable for P6/C4 chemistry. Using one SMRT cell on the PacBio RSII sequencing platform, 108,842 reads with a mean read length of 7,363 bp were obtained. The reads were assembled using Hierarchical Genome Assembly Process 3 (HGAP3) within the SMRT Analysis version 2.3.0 software with the default parameters^44^. The structure and functional annotation of protein-coding genes, including tRNA genes, were predicted using the RAST automatic annotation pipeline^45^. rRNA genes were identified with RNAmmer^46^.

### Selection and characterization of *Pseudomonas* strains

Sixteen *Pseudomonas* strains were selected for a comparative genome analysis based on their reported biological control properties. The newly sequenced *P. putida* 1A00316 was selected based on its extreme habitat and ability to kill *M. incognita*. The other 15 *Pseudomonas* strains were selected, in part, owing to their complete genome sequences and annotation information. In addition, among the seven *P. fluorescens* group strains, *P. fluorescens* F_113, *P. protegens* CHA0, and *P. protegens* Pf-5 are effective biological control agents against *M. incognita*. The genome sequences and annotation information for the 15 *Pseudomonas* strains were downloaded from NCBI (http://www.ncbi.nlm.nih.gov/, Table 1).

### Phylogenetic tree construction

A phylogenetic tree of 16 *Pseudomonas* strains was generated based on the concatenated sequences of 16S rRNA and five conserved housekeeping genes (*dnaE*, *guaA*, *gyrB*, *recA*, and *rpoB*) for each strain, and a multi-sequence alignment was generated using MUSCLE^47^. Gblocks was employed to identify conserved regions from which to generate the phylogenetic tree^48^. The phylogeny was constructed using MrBayes (GTR Substitution model, mcmc method, discard first 250 trees sampled) and visualized using TreeDyn^49^. In addition, 2,408 conserved single-copy proteins common to the 16 *Pseudomonas* genomes were identified with orthoMCL. After concatenation, cluastal omega^50^ was used to generate a multi-sequence alignment for these single-copy proteins and a phylogenetic tree was generated using Phylip (version 3.696) based on the maximum likelihood method.

### Identification of virulence factors

Virulence factors were identified based on the core dataset in VFDB database using BLASTP with an E-value cut-off of 1e^−5^ and an identity threshold of 60%^28^.

### Detection of protein families

An all-versus-all BLASTP search was used to identify similar proteins. After filtering proteins with low sequence quality (The length of these proteins is less than 10 and the percentage of stop codon is higher than 20%), homologous proteins and clustered protein families were identified using orthoMCL with the default parameters^29^.

### Purification and identification of a nematicidal factor as the alkaline metalloproteinase AprA

*P. putida* 1A00316 fermentation liquid (200 mL) was collected and centrifuged at 4225 × g for 15 min at 4 °C. The resultant supernatant was precipitated with ammonium sulfate to 60% saturation with a stirrer (4 °C). The precipitate was suspended in a minimum amount of sodium phosphate buffer (pH 7.4) and dialyzed against 50 mM phosphate buffer at 4 °C for 24 h with a stirrer.

The effect of temperature on nematicidal activity was examined after boiling the crude protein for 60 min, and cooling to room temperature. The effect of proteinase K was determined by mixing the crude protein with proteinase K (1 mg/mL) at 40 °C (pH 8.0). As a control, the crude protein was inactivated by heating at 100 °C and mixed with protease. Finally, the activity was quantitatively assayed as described below.

To further purify the crude protein, the solution was filtered through a membrane (0.22 μm), loaded onto a Sephadex G75 column connected to an ultraviolet detector, and eluted with ultrapure water. Fractions were collected and dried using a vacuum freeze dryer and then assayed by SDS-PAGE and by a nematicidal activity test as described below. Fractions with nematicidal activity were then loaded onto a CM52 column connected to an ultraviolet detector and the column was eluted with 10 mM phosphate buffer (pH 7.5) containing 0.5 M NaCl. The fractions were collected, dried as above, and then assayed again by SDS-PAGE and for nematicidal activity.

### Nematicidal activity bioassays *in vitro*

To examine nematicidal activity *in vitro*, 200 μL crude filtrate or fractions of the eluant from Sephadex or CM52 column were transferred to 96-well plates and then the wells were filled with freshly hatched juvenile suspension (approximately 30 *M. incognita*/μL). Each treatment was replicated three times. The plates were incubated at 20 °C for 48 h, and dead *M. incognita* was counted after exposure under an inverted microscope. *M. incognita* was considered dead when no movement was observed for 2 s after mechanical touching with a needle. The percentages of dead nematodes observed were corrected by eliminating the natural death in a negative control according to the Schneider-Orelli formula^51^.

### SDS-PAGE and MALDI-TOF MS

SDS-PAGE was performed with the Mini-PROTEAN III Gel System (Bio-Rad, Hercules, CA, USA) using 1.0-mm-thick slab gels of 12% (v/v) polyacrylamide. The proteins were stained with Coomassie Brilliant Blue G-250. Protein bands identified by Coomassie Brilliant Blue were excised and subjected to MALDI-TOF MS analyses.

### Analysis of key genes encoding 2,4-DAPG and HCN in *P. putida* 1A00316

The retention times of 2,4-DAPG in crude extracts of *P. putida* 1A00316 were compared by HPLC (Shimadzu, Kyoto, Japan). In addition, the *phlD* gene encoding 2,4-DAPG was PCR-amplified. The forward primer for *phlD* was 5′-ACCCACCGCAGCATCGTTTATGAGC-3′ and the reverse primer was 5′-CCGCCGGTATGGAAGATGAAAAAGTC-3′. The key gene encoding HCN was also confirmed by PCR amplification. The forward primer for the gene was 5′-GCCTGCTCGTTCAACCGTA-3′ and the reverse primer was 5′-CGCAGCCAGCCCACGTC-3′.

### Biochemical identification of HCN from *P. putida* 1A00316

HCN was detected according to the method in reference^52^. The indicator solution contained 38.46 mL of saturated picric acid solution (1.3%) and 61.54 mL of sodium carbonate solution (3.25%), mixed evenly. Filter paper was cut into triangles, dried after sterilization, and immersed in the indicator solution until saturated. After drying, the paper was used as a HCN indicator. *P. putida* 1A00316 was inoculated onto a Petri dish, the indicator paper was placed on the inside of the Petri dish cover, and the Petri dish was sealed. A Petri dish without *P. putida* 1A00316 was regarded as a negative control. After culturing at 28 °C for 3 to 4 days, the color of the indicator paper was observed. If the color of the indicator paper changed from yellow to orange or red, it was confirmed that *P. putida* 1A00316 can produce HCN.

### Extraction and purification of a new nematicidal compound in *P. putida* 1A00316

*P. putida* 1A00316 was cultivated for 48 h at 28 °C with shaking (180 rpm) in 500 mL Erlenmeyer flasks containing 200 mL of 2216E medium. The fermentation was centrifuged at 4225 × g for 10 min at 4 °C. The supernatant was extracted with equal volumes (100 mL) of *n*-butyl alcohol three times. The organic phase was evaporated, and the residue was dissolved in 2 mL of methanol and filtered through a 0.22 μm filter.

The crude extract was analyzed by chromatography using a silica gel column (5 × 120 cm) containing 160 g silica gel (200–300 mesh) eluted with a stepwise ethyl acetate/MeOH gradient of increasing polarity. The fractions were monitored by thin-layer chromatography (TLC, ethyl acetate/MeOH 6:1, spraying with iodine), and similar fractions were combined and tested against J2 *M. incognita*. Fractions that showed high nematicidal activity were loaded on a silica gel column, eluted with CH_2_Cl_2_:MeOH at various ratios (80:1, 50:1, and 30:1), screened by TLC (CH_2_Cl/MeOH 15:1, spraying with iodine) and again tested against J2 *M. incognita*. The active fraction was further purified by column (90 × 1.8 cm) chromatography on a Sephadex LH-20 (stepwise gradient of 50–100% methanol).

The supernatant was filtered with a 0.22 μm filter, and 10 μL samples were loaded for HPLC analysis by injection onto an Agilent TC-C18 column (4.6 × 250 mm, Santa Clara, CA, USA). Elution was performed with acetonitrile/sterile distilled water (SDW) (2:8, v/v). A variable-wavelength recorder was set at 210 nm to detect the compounds eluted from the column at a flow rate of 1.1 mL min^−1^.

### Identification of a new nematicidal compound in *P. putida* 1A00316

The chemical structures of isolated compounds were determined by electrospray ionization mass spectrometry (ESI) and ^1^H and ^13^C NMR. Chromatographic separation was performed on an Agilent 6540 UHD Accurate-Mass Q-TOF LC/MS, and chromatographic analysis was achieved on a C18 column (particle size 5 mm, 100 × 2.1 mm, Agilent Technology) with an injection volume of 1 μL. The mobile phase was acetonitrile/SDW (2:8, v/v) at a flow rate of 0.3 mL/min. Approximately 5 mg of the purified compound was dissolved in methanol-*d*_4_ (CD_3_OD) and subjected to a spectral analysis. NMR spectra were recorded on a Bruker DRX 500 NMR instrument, operated at 500 MHz for ^1^H NMR, and 125 MHz for ^13^C NMR, both at room temperature. ^1^H and ^13^C NMR assignments were supported by ^1^H-^1^H correlation spectroscopy (COSY), heteronuclear multiple-quantum coherence (HMQC), nuclear Overhauser effect spectrometry (NOESY), and heteronuclear multiple-bond correlation (HMBC) experiments.

### Determination of the absolute configuration of the nematicidal compound in *P. putida* 1A00316

The absolute configurations of the amino acids in compounds were determined using Marfey’s FDAA (1-fluoro-2, 4-dinitrophenyl-5-l-alanine amide) derivatization method^53^. A sample (1 mg) of the compound was heated with 0.1 mL of 6N HCl at 120 °C for 20 h. The hydrolysate was evaporated until dry and dissolved in H_2_O. The retention times of all derivatives obtained from the hydrolysis of test compounds were compared with the derivatized standard d-amino and l-amino acids.

## Additional Information

**How to cite this article**: Guo, J. *et al.* Comparative genomic and functional analyses: unearthing the diversity and specificity of nematicidal factors in *Pseudomonas putida* strain 1A00316. *Sci. Rep.* **6**, 29211; doi: 10.1038/srep29211 (2016).

## Supplementary Material

Supplementary Information

## Figures and Tables

**Table 1:** Features of 16 *Pseudomonas* genomes.

Features	Chromosome size (Mb)	G + C (%)	CDSs	Average CDSs length (nt)	Coding (%)	rRNA genes	tRNA genes	Scaffolds	Accession number	Virulence factors
Pf_A506	5.96	60.0	5267	993	87.7	19	69	1	NC_017911.1	87
Pps_CHA0	6.87	63.4	6115	996	88.6	15	68	1	NC_021237.1	101
Pf_F113	6.85	60.8	5862	1011	86.6	16	66	1	NC_016830.1	110
Pf_Pf0-1	6.44	60.5	5722	1008	89.6	19	73	1	NC_007492.2	77
Pps_Pf-5	7.07	63.3	6108	1013	87.5	16	71	1	NC_004129.6	106
Pf_Q2–87	6.37	60.6	5597	996	87.6	19	68	1	NZ_CM001558.1	103
Pf_SBW25	6.72	60.5	5921	1000	88.1	16	66	1	NC_012660.1	91
Pp_1A00316	5.72	64.4	5039	987	87.2	22	76	1	CP014343	92
Pp_BIRD-1	5.73	61.7	4960	1002	86.7	22	64	1	NC_017530.1	64
Pp_F1	5.96	61.9	5250	1005	88.5	19	76	1	NC_009512.1	66
Pp_GB-1	6.08	61.9	5408	1003	89.2	22	74	1	NC_010322.1	66
Pp_H8234	6.87	61.6	6357	934	86.4	18	69	1	NC_021491.1	72
Pp_HB3267	5.88	62.6	5292	966	87.0	22	70	2	NC_019905.1	69
Pp_KT2440	6.18	61.5	5350	1000	86.5	22	74	1	NC_002947.3	63
Pp_S16	5.98	62.3	5218	971	84.7	19	70	1	NC_015733.1	65
Pp_W619	5.77	61.4	5182	988	88.7	22	75	1	NC_010501.1	62

**Table 2:** NMR results for the nematicidal compound in *P. putida* 1A00316.

Peak	^1^H	Multiplicity	J-value	C^13^
1	–	–	–	172.4
2	4.2	m	–	60.2
3	2.33	m	–	29.8
	1.93	m	–	
4	1.94	m		23.3
	2.03	m		
5	3.55	m	–	46.4
1’	–	–	–	167.7
2’	4.07	t	2.21	61.6
3’	2.16	m	–	37.4
			–	
4’	1.32	m	–	25.7
	1.44	m	–	
5’		t	7.46	12.6
6’		d	7.18	15.7

**Table 3:** Nematicidal activity for cyclo-(l-isoleucyl-l-proline) at different concentrations.

Concentration (μg/mL)	Mortality (%)		
	48 h	72 h	96 h
130	12.07 ± 2.12	28.98 ± 4.03	29.90 ± 1.34
330	28.01 ± 4.76	34.17 ± 1.52	43.00 ± 2.86
500	37.50 ± 2.29	46.19 ± 2.76	50.00 ± 3.03
CK	2.05 ± 0.36	5.70 ± 0.76	7.90 ± 1.02

CK (control check): sterile water.
