# Supplementary material for: Comparative genomic and functional analyses: unearthing the diversity and specificity of nematicidal factors in Pseudomonas putida strain 1A00316
Source: Sci Rep. 2016 Jul 7;6:29211. doi: 10.1038/srep29211 (PMC4935845; doi:10.1038/srep29211)
Supplement: Supplementary Information [file srep29211-s1.pdf]

Supplementary information for

Comparative genomic and functional analyses: unearthing the diversity and specificity of nematicidal factors in *Pseudomonas putida* strain 1A00316

Jing Guo[1,2§], Xueping Jing[1§], Wen-Lei Peng[2§], Qiyu Nie[1§], Yile Zhai[1], Zongze Shao[3], Longyu Zheng[1], Minmin Cai[1], Guangyu Li[3], Huaiyu Zuo[1], Zhitao Zhang[1], Rui-Ru Wang[2], Dian Huang[1], Wanli Cheng[1], Ziniu Yu[1], Ling-Ling Chen[2]*, Jibin Zhang[1]*

[1]State Key Laboratory of Agricultural Microbiology and National Engineering Research Center of Microbe Pesticides, College of Life Science and Technology, Huazhong Agricultural University, Wuhan 430070, Hubei, China

[2]Agricultural Bioinformatics Key Laboratory of Hubei Province, College of Informatics, Huazhong Agricultural University, Wuhan 430070, Hubei, China

[3]Key Laboratory of Marine Biogenetic Resources, Third Institute of Oceanography, State Oceanic Administration, Xiamen 361005, Fujian, China

***Correspondence:**

Jibin Zhang, State Key Laboratory of Agricultural Microbiology, College of Life Science and Technology, Huazhong Agricultural University, Wuhan 430070, Hubei, China.

E-mail: zhangjb@mail.hzau.edu.cn

Fax: 86-27-87287254; Tel: 86-27-87287701

Ling-Ling Chen, Agricultural Bioinformatics Key Laboratory of Hubei Province, College of Informatics, Huazhong Agricultural University, Wuhan 430070, Hubei, China

E-mail: llchen@mail.hzau.edu.cn

Tel (Fax): 86-27-87280877

[§] **Equal contributors**

Figure S1. Maximum likelihood phylogeny inferred from 2,408 single-copy conserved protein-coding genes in the *P. putida* 1A00316 genome.

Figure S2. Sequence alignment of the AprA protein in various *Pseudomonas* strains. Green sequence fragments are the ZnMc_serralysin_like domain, and red amino acids are active sites.

Figure S1

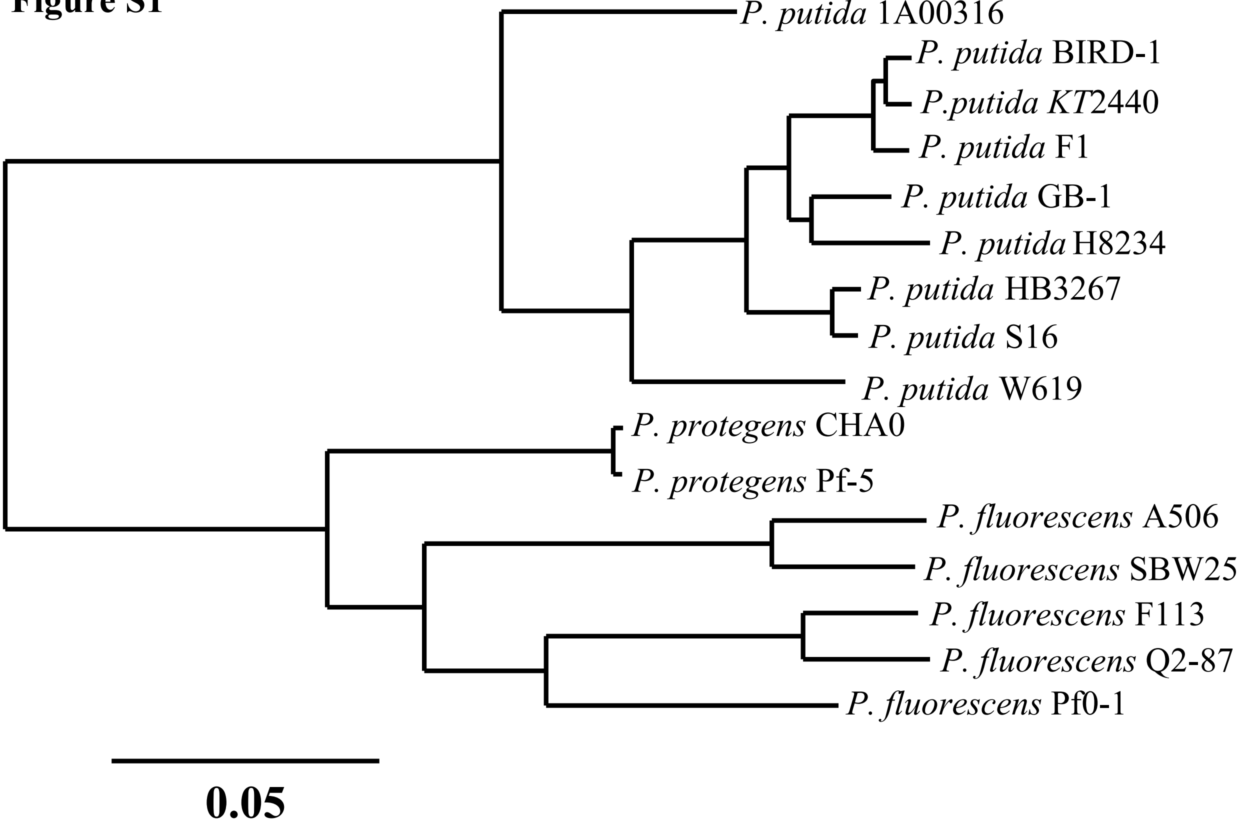

P. putida 1A00316
P. putida BIRD-1
*P.putida KT*2440
P. putida F1
P. putida GB-1
P. putida H8234
P. putida HB3267
P. putida S16
P. putida W619
P. protegens CHA0
P. protegens Pf-5
P. fluorescens A506
P. fluorescens SBW25
P. fluorescens F113
P. fluorescens Q2-87
P. fluorescens Pf0-1
0.05

Figure S2

```
            ....|....| ....|....| ....|....| ....|....| ....|....| ....|....| ....|....| ....|....| ....|....| ....|....|
                   10         20         30         40         50         60         70         80         90        100
Pp_1A00316  MSKVKENAIV SAVSALQP --   ---KGPSSSF GLIDSFAHQY DRGG-ANING KKSFTADQAA DHILRDGAAW KDLN       KDGTIS LTYTFLTKAP -SDFYSRGLG
Pps_CHA0    MSKVTENAIA SAEQQLVP --    --  LAAASSAY NQINSFSHQY DRGGNLTVNG KPSFSVDQAA TQLLRDGAAY KDLN   GNGKID LTYTFLTSAS SSTMYKHGIS
Pps_Pf-5    MSKVTENAIA SAEQQLVP --    --  LAAASSAY NQINSFSHQY DRGGNLTVNG KPSFSVDQAA TQLLRDGAAY KDLN   GNGKID LTYTFLTSAS SSTMYKHGIS
Pf_F113     MSKVKSSAID SAEQLLWAPQ S-LAAPSNAF NQINSFSHQY DRGGNLTVNG KPSYSVDEAA TQLLRDGAAY QDRD      GSGKIE LTYTFLTSAS SSTMNKHGIS
Pf_Q2-87    MSKVKNSAID SSEQLLWAPQ S-LAASSNAF NQINSFSHQY DRGGNLTVNG KPSYSVDQAA TQLLRDGAAY QDRD      GSGKIE LTYTFLTSAS SSTMNKHGIS
Pf_A506     MSKVKDKAIV SA-------- ---AQASTAY SQIDSFSHLY DRGGNLTVNG KPSYTVDQAA TQLLRDGAAY RDFD      GNGKID LTYTFLTSAT QSTMNKHGIS
Pf_SBW25    MSKVKDKAIV SA-------- ---AQASTAY SQIDSFSHLY DRGGNLTING KPSYTVDQAA TQLLRDGATY RDFD      GNGKID LTYTFLTSAS SSTMNKHGIS
Pf_Pf0-1    MSKVKANAVD TAEQAFQLNA APLAASSSAF NQINSFSHQY DRGGNLTVNG KPSFSVDQAA TQLLRDGAAY QDKD      GSGKIE LTYTFLTSAS SSTMYKHGIS
```

```
            ....|....| ....|....| ....|....| ....|....| ....|....| ....|....| ....|....| ....|....| ....|....| ....|....|
                  110        120        130        140        150        160        170        180        190        200
Pp_1A00316  SFSQFSDLQK QQAKLSMQSW ADVAKVTFTE AASGGDGHMT FGNFSAS- NG GAAFAYLPFD GPGSHKGESW YLINSGYQVN INPGTGNYGR QTLT  HEIG HV
Pps_CHA0    GFSQFSAQQK AQAVLAMQSW ADVANVVFTE KASGGDAHMT FGNYSGGQDG AAAFAYLPGT GAG-YDGTSW YLINSSYTQN KNPDLNNYGR QTLT  HEIG HT
Pps_Pf-5    GFSQFSAQQK AQAVLAMQSW ADVANVVFTE KASGGDAHMT FGNYSGGQDG AAAFAYLPGT GAG-YDGTSW YLINSSYTQN KNPDLNNYGR QTLT  HEIG HT
Pf_F113     GFSPFSAQQK GQATLAMQSW ADVANVTFTE KASGGDGHMT FGNYSSGQDG AAAFAYLPGT GAG-YDGTSW YLINSSYTQN KNPDLNNYGR QTLT  HEIG HS
Pf_Q2-87    GFSQFSAQQK GQAALAMQSW ADVANVTFTE KASGGDGHMT FGNYSGGQDG AAAFAYLPGT GAG-YDGTSW YLINSSYTQN KNPDLNNYGR QTLT  HEIG HT
Pf_A506     GFSQFNTQQK AQAALAMQSW ADVANVTFTE KASGGDGHMT FGNYSSGQDG AAAFAYLPGT GAG-YDGTSW YLTNNSYTPN KTPDLNNYGR QTLT  HEIG HT
Pf_SBW25    GFSQFNAQQK AQAVLAMQSW ADVANITFTE KASGGDAHMT FGNYSGGQDG AAAFAYLPGT GAG-YDGTSW YLTNNSYTPN KTPDLNNYGR QTLT  HEIG HT
Pf_Pf0-1    GFSQFSTQQK AQAVLAMQSW ADVANVTFTE KASGGDGHMT FGNYSGGQDG AAAFAYLPGT GAG-YDGTSW YLINSGYTQN KNPDLNNYGR QTLT  HEIG HT
```

```
            ....|....| ....|....| ....|....| ....|....| ....|....| ....|....| ....|....| ....|....| ....|....| ....|....|
                  210        220        230        240        250        260        270        280        290        300
Pp_1A00316  LGLS HPGDYN AGEGNPTYRD ADYAQDTRGY SVMSYWSESN NGQNFIKGGG QYYASAPLMD DILAIQKLYG ANYATRASDT TYGFNSTADR DFYSATSASS
Pps_CHA0    LGLAHPGDYN AGEGNPSYKD ASYGQDTRGY SVMSYWSESN TSQNFSKGGV EAYSSGPLMD DIAAIQKLYG ANYSTRAGDT TYGFNSNTGR DFYSASSSSD
Pps_Pf-5    LGLAHPGDYN AGEGNPSYKD ASYGQDTRGY SVMSYWSESN TSQNFSKGGV EAYSSGPLMD DIAAIQKLYG ANYSTRAGDT TYGFNSNTGR DFYSASSSSD
Pf_F113     LGLAHPGDYN AGEGSPTYDD ATYGQDTRGY SLMSYWSESN TDQNFSKGGV EAYASGPLMD DIAAIQKLYG ANTTTRTGDS TYGFNSNTGR DFLSASSSSD
Pf_Q2-87    LGLAHPGDYN AGEGSPTYDD ATYGQDTRGY SVMSYWSESN TSQNFSKGGV EAYSSGPLMD DIAAIQKLYG ANTTTRTGDT TYGFNSNAGR DFLSASSSSD
Pf_A506     LGLAHPGDYN AGNGNPTYND ATYGQDTRGY SLMSYWSESN TNQNFSKGGV EAYASGPLID DIAAIQKLYG ANLSTRATDT TYGFNSNTGR DFLSASSNAD
Pf_SBW25    LGLAHPGDYN AGEGAPTYND ATYGQDTRGY SLMSYWSESN TNQNFSKGGV EAYASGPLID DIAAIQKLYG ANYNTRAGDT TYGFNSNTGR DFLSATSNAD
Pf_Pf0-1    LGLAHPGDYN AGNGNPTYND ATYGQDTRGY SVMSYWSESN TSQNFSKGGV EAYSSGPLMD DIAAIQKLYG ANLTTRTGDT TYGFNSNAGR DFLSASSSAD
```

```
            ....|....| ....|....| ....|....| ....|....| ....|....| ....|....| ....|....| ....|....| ....|....| ....|....|
                  310        320        330        340        350        360        370        380        390        400
Pp_1A00316  KLVFSVWDGG GNDTFDFSGF TQNQKINLNE ASFSDVGGMV GNVSIAKGVT IENAIGGSGN DLLIGNALAN VLKGGAGNDI IYGGGGADQL WGGSGSDTFV
Pps_CHA0    KLVFSVWDGG GNDTLDFSGF TQNQKINLNE ASFSDVGGMV GNVSIAQGVT VENAIGGSGN DLLIGNNAAN VLKGGAGNDI IYGAGGADQL WGGSGSDTFV
Pps_Pf-5    KLVFSVWDGG GNDTLDFSGF TQNQKINLNE ASFSDVGGMV GNVSIAQGVT VENAIGGSGN DLLIGNNAAN VLKGGAGNDI IYGAGGADQL WGGSGSDTFV
Pf_F113     KVVFSVWDAG GRDTLDFSGF TQNQKINLND ASFSDVGGMV GNVSIAQGAV IENAIGGSGS DLLIGNTVAN ELKGGAGNDI LWGAGGADKL WGGSGSDTFV
Pf_Q2-87    KVVFSVWDAG GRDTLDFSGF TQNQKINLND ASFSDVGGMV GNVSIAKGAV IENAIGGSGS DLLIGNAIAN ELKGGAGNDI LWGAGGADKL WGGSGSDTFV
Pf_A506     KLVFSVWDGG GNDTLDFSGF TQNQKINLTA TSFSDVGGLV GNVSIAKGVT IENAFGGSGN DLIIGNQVAN TIKGGAGNDL IYGGGGADQL WGGTGSDTFV
Pf_SBW25    KLVFSVWDGG GNDTLDFSGF TQNQKINLNE ASFSDVGGLV GNVSIAKGVT IENAFGGAGN DLIIGNNAAN IIKGGAGNDL IYGGGGADQL WGGAGNDTFV
Pf_Pf0-1    KLVFSVWDAG GKDTFDFSGF TQNQKINLNE ASFSDVGGLV GNVSIAKGVT IENAIGGSGN DLLIGNSAAN ELKGGAGNDI IWGAGGADKL WGGAGSDTFV
```

```
            ....|....| ....|....| ....|....| ....|....| ....|....| ....|....| ....|....| ....|....| ....|....|
                  410        420        430        440        450        460        470        480        490
Pp_1A00316  FAAVSDSTKA APDRIMDFTS GVDKIDLSAI SAFAVNKLPL QFVNAFTGHA GEALLTYDQA TNLGSLAIDF TGNSSADFLV TTVGQAAVTD IVV
Pps_CHA0    FAASTDSKPG AADQIMDFVS GLDKIDLTGI TKGAG----L HFVNAFTGAA GDAILTTSGG VST       --  LSVDF SGHGVADFLV STVGQAAVSD IVA
Pps_Pf-5    FAASTDSKPG AADQIMDFVS GLDKIDLTGI TKGAG----L HFVNAFTGAA GDAILTTSGG VST       --  LSVDF SGHGVADFLV STVGQAAVSD IVA
Pf_F113     FAASSDSRPG ATDQILDFVS GLDKIDLTGI TKGAG----L HFVSAFTGAV GDAVLSTSGG NSL       --  LSVDF SGQGVADFLV STVGQAAVSD IVA
Pf_Q2-87    FAASSDSRPG ATDQILDFAS GLDKIDLTGI TQGAG----L HFVNAFTGAA GDAVLSTSGG NSL       --  LSVDF SGHGVADFLV STVGQAAVSD IVA
Pf_A506     YGASSDSRPG AADKIFDFTS GSDKIDLSGI TKGAG----V TFVNAFTGHA GDAVLTYASG TNLGTLAVDF SGHGVADFLV TTVGQAAASD IVA
Pf_SBW25    FGASSDSKPG ASDKIFDFTS GSDKIDLSGI TKGAG----L TFVNAFTGHA GDAVLTYAAG TNLGTLAVDF SGHGVADFLV TTVGQAAVSD IVA
Pf_Pf0-1    FAASSDSKPG AIDQILDFVS GLDKIDLTGI TNGAG----L HFVSAFTGAA GDAVLTSSGG NSL       --  LSVDF SGHGVADFQV STVGQAATSD IVA
```
